# Preliminary study of clinical application on IMRT three‐dimensional dose verification‐based EPID system

**DOI:** 10.1002/acm2.12098

**Published:** 2017-06-08

**Authors:** Miaoyun Huang, David Huang, Jianping Zhang, Yuangui Chen, Benhua Xu, Lixin Chen

**Affiliations:** ^1^ Department of Radiation Oncology Fujian Medical University Union Hospital Fuzhou China; ^2^ Medical Physics Graduate Program Duke Kunshan University Kunshan China; ^3^ State Key Laboratory of Oncology in South China Sun Yat‐sen University Cancer Center Guangzhou China

**Keywords:** Delta4, electronic portal imaging system (EPID), intensity‐modulated radiation therapy, quality assurance, three‐dimensional dose verification

## Abstract

The three‐dimensional dose (3D) distribution of intensity‐modulated radiation therapy (IMRT) was verified based on electronic portal imaging devices (EPIDs), and the results were analyzed. Thirty IMRT plans of different lesions were selected for 3D EPID‐based dose verification. The gamma passing rates of the 3D dose verification‐based EPID system (Edose, Version 3.01, Raydose, Guangdong, China) and Delta4 measurements were then compared with treatment planning system (TPS) calculations using global gamma criteria of 5%/3 mm, 3%/3 mm, and 2%/2 mm. Furthermore, the dose–volume histograms (DVHs) for planning target volumes (PTVs) as well as organs at risk (OARs) were analyzed using Edose. For dose verification of the 30 treatment plans, the average gamma passing rates of Edose reconstructions under the gamma criteria of 5%/3 mm, 3%/3 mm, and 2%/2 mm were (98.58 ± 0.93)%, (95.67 ± 1.97)%, and (83.13 ± 4.53)%, respectively, whereas the Delta4 measurement results were (99.14% ± 1.16)%, (95.81% ± 2.88)%, and (84.74% ± 7.00)%, respectively. The dose differences between Edose reconstructions and TPS calculations were within 3% for D_95%_, D_98%_, and D_mean_ in each PTV, with the exception that the D_98%_ of the PTV‐clinical target volume (CTV) in esophageal carcinoma cases was (3.21 ± 2.33)%. However, the larger dose deviations in OARs (such as lens, parotid gland, optic nerve, and spinal cord) can be determined based on DVHs. The difference was particularly obvious for OARs with small volumes; for example, the maximum dose deviation for the lens reached (−6.12 ± 5.28)%. A comparison of the results obtained with Edose and Delta4 indicated that the Edose system could be applied for 3D pretreatment dose verification of IMRT. This system could also be utilized to evaluate the gamma passing rate of each treatment plan. Furthermore, the detailed dose distributions of PTVs and OARs could be indicated based on DVHs, providing additional reliable data for quality assurance in a clinic setting.

## 1 INTRODUCTION

Compared with three‐dimensional conformal radiotherapy (3DCRT), intensity‐modulated radiotherapy (IMRT) has steep dose gradients. Many factors can affect the dose accuracy in IMRT delivery, and these include (a) the leaf position, speed, and sequencing algorithm of the multileaf collimator (MLC), (b) the flatness and symmetry of the accelerator beam profile, (c) the accuracy of the physical model of the treatment planning system (TPS), and (d) more segments and variations in time and space of the dose rate. Thus, the implementation of efficient and safe quality assurance (QA) for IMRT is indispensable for ensuring that the plan dose can be accurately delivered to a patient.

The traditional approach for verifying an IMRT plan is mainly done with two‐dimensional (2D) or three‐dimensional (3D) detectors based on a uniform phantom, such as a film, graphic matrix, or electronic portal imaging system (EPID).^1^, ^2^, ^3^, ^4^, ^5^, ^6^ However, the dose variation cannot be obtained solely through model‐based dose verification due to the lack of patient's anatomic information. Increasingly, researchers have studied the feasibility of 3D dose verification using the patient anatomy combined with a calculation model.^7^, ^8^, ^9^, ^10^ Studies have shown that 3D dose verification could provide more information than 2D dose verification, such as the gamma passing rate, DVH, and the dose distributions in axial, coronal, and sagittal views.

EPIDs are widely used in pretreatment QA for IMRT due to their advantages of a large detection area, high resolution, rapid measurement, convenient operation, few angle response characteristics, and no angular dependence.^10^, ^11^, ^12^, ^13^, ^14^, ^15^ Based on these advantages, the EPID‐based method for 3D dose verification appears to be a promising direction for IMRT and volumetric‐modulated arc therapy (VMAT) dose verifications. The IMRT 3D dose verification system described in this manuscript is an EPID‐based 3D dose reconstruction verification system (Edose, Version 3.01, Raydose, Guangdong, China) that assessed the coincidence between the delivered and planned doses. The Edose system applies the pixel values of the EPID images as input parameters, and the images are then convolved and deconvolved with energy deposition kernel from photons to reconstruct the fluence map of the actually delivered beam. The fluence map of the delivered beam then combines with collapsed‐cone convolution/superposition (CCCS) to calculate the CT image‐based 3D dose distributions. The advantage of Edose system is that it provides patient anatomy‐based 3D dose verification and can thus determine the delivery errors of treatment plans and provide dose–volume information for planning target volumes (PTVs) and organs at risk (OARs). A detailed description of the Edose QA system is provided in Part II. Because the Edose system constitutes a novel device using new technology for IMRT pretreatment dose verification, it was necessary to ensure its accuracy in clinical application. This study therefore aimed to evaluate the accuracy of the Edose system by comparing its reconstructed results with those measured using more established methods, namely the Delta4 device.^16^, ^17^, ^18^ A total of 30 IMRT treatment plans are evaluated in the clinical application study.

## 2 METHODS AND MATERIALS

### 2.A Measurement devices

The measurements were performed using the Varian Trilogy (Varian Medical Systems, Palo Alto, CA, USA) LINAC with 120 multileaf collimators (MLCs). The projected leaf width at the isocenter is 0.5 cm in the inner 20 cm and 1 cm in the outer 20 cm. The accelerator was equipped with an aS1000 EPID (Varian Medical Systems) with an effective detection area of 40 cm × 30 cm, 1024 × 768 pixels dimension and adjacent detectors with a pixel size of 0.39 mm. The Delta4 device (Version 2013 February, Scandidos, Uppsala, Sweden) is a cylindrical polymethyl methacrylate phantom with a diameter of 22 cm and a length of 40 cm. The device is composed of two crossing orthogonal diode arrays that consisted of three detection boards. One of the detector boards passes through the entire diameter of the phantom, and the other two wing detector boards are separated to allow the main detector board to pass between them. The Delta4 device has 1069 cylindrical diodes and a 20 cm × 20 cm detection area. The diodes are spaced at 0.5 cm intervals in the central 6 cm × 6 cm of the planes and at 1 cm intervals over the remaining area of the central 20 cm × 20 cm of the planes. Each diode has an area of 0.78 mm^2^.

### 2.B The theory of 3D dose reconstruction in the Edose system

The Edose system is a QA tool based on the patients' anatomy. This system uses the pixel values of images captured by EPID from treatment fields in air without a phantom/patient as input parameters. The images are then reconstructed into a fluence map of the actually delivered beam through deconvolution and convolution. The CCCS algorithm was used for the 3D dose calculations and the 3D gamma evaluations. The theoretical formulas^19^, ^20^ of the fluence maps were reconstructed from the EPID image in the Edose system as follows:

(1) $$D_{ij}=a\cdot P_{ij}^{}{⊗}^{-1}K_{1}\left(d_{ij}\right)⊗K_{2}\left(d_{ij}\right)\cdot f\left(r_{ij}\right)$$

where

(2) $$K_{1}\left(d_{ij}\right)=\left(1-c\right)\exp\left(-\mu_{1}d_{ij}\right)+c\exp\left(-\mu_{2}d_{ij}\right)$$

(3) $$K_{2}\left(d_{ij}\right)=\text{exp}\left(-\mu_{3}d_{ij}\right)$$

(4) $$f\left(r_{ij}\right)=1+c_{r}\exp\left[-{\left(r_{ij}-\varepsilon \right)}^{2}/\left(2\sigma^{2}\right)\right]$$

where “*a*”, which is proportional to monitor units (MU), is a coefficient denoting the EPID pixel values, “*P*_ij_” denotes the EPID image pixel values, and “*K*_1_(*d*_ij_)” is the EPID's scattering kernel and represents the energy scattering distribution after the interaction of the incident photon with EPID. In addition, *μ*_1_ and *μ*_2_ are the attenuation coefficients, which depend on the energy and the materials, c is the ratio constant, “*K*_2_(*d*_ij_)” is the fuzzy convolution kernel, which is the boundary factor for depicting the penumbra, *μ*_3_ affects the gradient of the penumbra, “ƒ(r_ij_)” is the Gaussian distribution function, which is introduced for shape correction, and *ε*,* σ*, and *C*_r_ are obtained by comparing the profile after the reconstruction, mainly aimed at the large field to execute the adjustment. Furthermore, these three parameters reflect the upward curve of the saddle‐shaped part of the profile. The variables “*K*_2_(*d*_ij_)” and “ƒ(r_ij_)” can correct the profile shape at various depths. *c*,* μ*_1_, *μ*_2_, *μ*_3_, *C*_r_, *ε*, and *σ* are parameters for the detector kernel and were determined using the central point doses, which were measured in air for fields of 3 cm × 3 cm to 25 cm × 25 cm using an ionization chamber.

The parameters were constants once the fit procedure was completed as a result of the invariant structure of EPID. The EPID‐based fluence maps for fields of 3 cm × 3 cm to 25 cm × 25 cm were compared with the ionization chamber results. The total scatter factor (S_c,p_) and the dose profiles were measured using an ionization chamber with a buildup cap in air by scanning an empty 3D water tank (MP3, PTW, Germany) at an SAD of 100 cm. The values of the parameters were determined when the deviation between the ion chamber measurements and the EPID‐based fluence maps was less than 2%. The values of the parameters applied in this study were *c* = 0.00013, *μ*_1_ = 11.3000, *μ*_3_ = 15.000, *C*_*r*_ = 0.028, *ε* = 6.000, and *σ* = 5.000.

### 2.C Clinical applications and evaluation of Edose

Prior to evaluation, comprehensive tests and evaluations to the Edose system were performed using the film and ionization chamber (IC) with uniform and human phantoms. The ionization chamber and radiochromic film were selected for measuring the point and planar doses for the single square and its combined fields and IMRT plans, and the corresponding results were compared to those reconstructed using Edose. The results showed that the point dose measured by the Edose agreed within 0.5% with the ionization chamber measurement in a uniform phantom. A minimum gamma pass rate of 95% was achieved for the comparison between Edose reconstructed dose maps and the planned dose maps when using the dose difference criterion of 5% of the maximum dose and a distance‐to‐agreement criterion of 3 mm (henceforth referred to as 5%/3 mm) and 3%/3 mm gamma criteria. The details are provided in our previously published manuscript.^21^ In the present study, 3D dose reconstruction results obtained using the Edose system and measured with Delta4 were compared with those calculated by TPS to verify the feasibility of the Edose system. Thirty IMRT patient plans, including 10 nasopharyngeal carcinoma (NPC) plans, 10 esophageal carcinoma (EPC) plans, and 10 rectal cancer (REC) plans, were selected randomly for the 3D dose verification of Edose.

#### 2.C.1 Treatment plan

Thirty IMRT patient plans were optimized and calculated using an Eclipse treatment planning system (Eclipse 10.0.42, Varian Medical Systems, USA). The dose was calculated using an analytical anisotropic algorithm (AAA) (Version 10.0.28) with a grid size of 2.5 mm and a dose rate of 400 MU/min. The NPC, EPC, and REC plans were designed with seven, five, and five fields, respectively. In addition, a 6‐MV photon was selected in all plans, and the MLC movement pattern used in this study was a sliding window. All of the IMRT plans were created based on the patient's anatomy, which was exported to the Varian Trilogy and Edose systems through the Aria10 network (Varian Medical Systems). The files exported to the Edose system included CT images, the treatment plan, the treatment dose, and anatomical structures.

The treatment plans were recalculated with Delta4 without changing the monitor units and were then delivered to the Delta4 phantom.

#### 2.C.2 Methods of measurements

The panel of EPID detectors was positioned at the isocenter with a source‐to‐detector distance (SDD) of 100 cm without any attenuating material in between. The pixel values of the EPID images of actual treatment fields were measured with an EPID device in integrated mode. The images were obtained with image acquisition system software (IAS3a, Varian, USA). The Edose system used the EPID‐based fluence maps to reconstruct the dose distributions. The corresponding dark and flood fields of the EPID were obtained prior to the measurement to correct for the background signal and the probe uniformity produced by the electronic components of the EPID. The images measured by the EPID were exported to the Edose system to perform the 3D dose reconstruction, and the reconstruction results were then compared with those obtained with the TPS.

Dose verification of all treatment plans was also performed with the Delta4 diode array cylindrical phantom. The center of the phantom was placed at a source‐axis distance (SAD) of 100 cm during the measurement. The phantom was calibrated relatively and absolutely for a 6‐MV beam according to the manufacturer's manual. Data analysis was performed with Scandidos software (Scandidos, Uppsala, Sweden), which allows the user to compare the measured dose distribution for a complete treatment plan with the dose distribution predicted by the TPS. The ambient temperature was entered into the Delta4 software prior to every measurement because the diode detector response varied with temperature.

#### 2.C.3 Evaluation methods

An analytical method for 3D gamma evaluations 22 was applied to compare the 3D dose distributions that were reconstructed using Edose and measured using Delta4 with those obtained with the TPS. A dose difference criterion of 5% of the maximum dose and a distance‐to‐agreement criterion of 3 mm were selected. A 3%/3 mm criterion and a more rigid 2%/2 mm criterion were also evaluated. The gamma evaluation was global for both devices, and the threshold used for the dose analysis was more than 10% of the prescribed dose. The same areas that were used for the dose comparisons were defined as regions of interest (ROIs). A graphics processing unit (GPU) operation was employed in the system to improve the efficiency of the 3D gamma calculation. 23, 24

Dose–volume histograms (DVHs) for both target areas and OARs were also compared between the TPS and the Edose system. The target areas were set as the PTVs, which were created by a 5 mm additional margin to the gross tumor volumes (GTVs) or clinical target volumes (CTVs). The CTV was calculated as the GTV plus 5–10 mm depending on the anatomical boundaries. CTV‐N‐L (or R) represents left (or right) of lymph nodes with a low risk of occult metastases. Furthermore, CTV1 was defined as lymph nodes with a high risk of occult metastases, and GTV‐N‐L (or R) corresponds to the left (or right) GTV of the neck lymph nodes. More information regarding the abbreviation of the target volumes is shown in Fig. 4.

For the target areas (PTVs), dose deviations of D_95%_ (∆D_95_), D_98%_ (∆D_98_), and D_mean_ (∆D_mean_) were selected for analyzing the dose deviation. Here, D_xx%_ denotes the dose up to xx% of the target volume, and the differences are expressed as the means ± one standard deviation (SD). The dose deviations are expressed (∆D_xx_) as (EPID D_xx%_‐TPS D_xx%_)/TPS D_xx%_ × 100%. The dose of OARs was analyzed with the maximum doses (D_max_) and dose–volume parameters V_x_ (the volume of the OAR that received a dose of x Gy). As an example, the maximum doses for the spinal cord, lens, optic nerve, and bowel were analyzed; V_5_ and V_20_ analyses were performed in the lungs; ∆D_mean_ and V_30_ analyses were performed in the parotids; ∆D_mean_ and V_40_ analyses were performed in the bladder; and ∆D_mean_ and V_20_ analyses were performed in the femoral heads.

## 3 RESULTS

### 3.A Gamma passing rate

**Figure 1:**
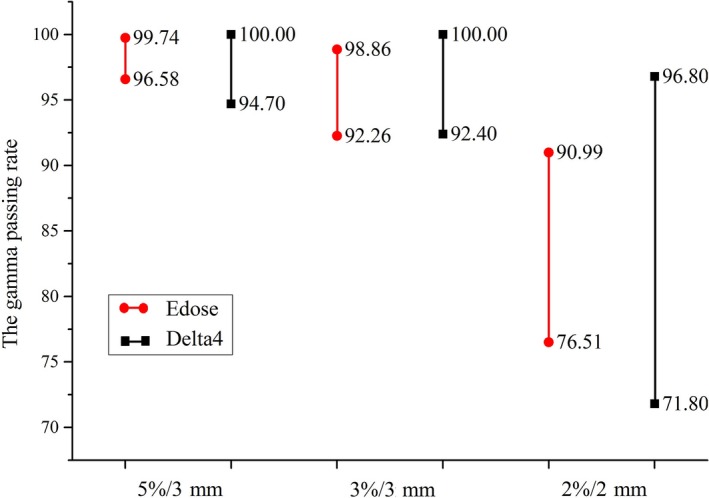
Range of the gamma passing rates obtained for 30 selected cases under three criteria using Edose and Delta4.

The gamma passing rates obtained through the Edose reconstructions and Delta4 measurements were compared with the TPS calculations. Figure 1 shows the range of the gamma passing rates of 30 selected cases at the gamma criteria of 5%/3 mm, 3%/3 mm and 2%/2 mm. The narrow range showed the good reproducibility and stability of the response obtained using the Edose system. Overall, the range of the gamma passing rate detected with Delta4 was greater than that detected with Edose.

**Figure 2:**
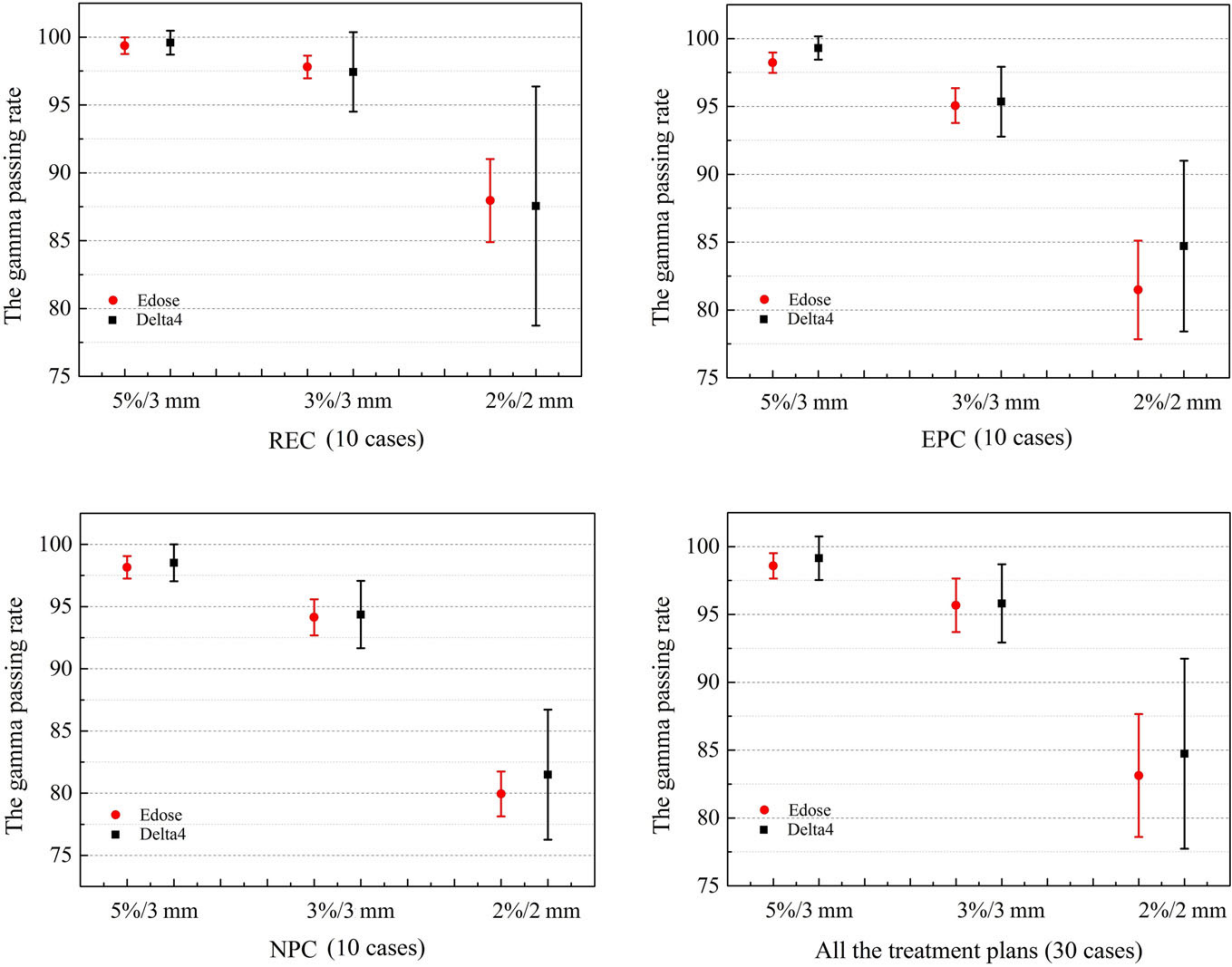
Comparison of the average gamma passing rates for the 30 IMRT cases obtained from Edose and Delta4 measurements. The red circle and the black box represent the mean values of the gamma passing rates obtained using Edose and Delta4, respectively, and the error bars represent the SDs of the corresponding gamma passing rates.

Figure 2 illustrates the gamma passing rates for the REC, EPC, and NPC plans as well as all 30 plans under the three above‐mentioned gamma criteria. The comparison results are expressed as the means ± SD in Fig. 2 and Table 1. As illustrated in Fig. 2, a similar gamma passing rate for NPC and EPC was obtained under the 5%/3 mm criterion. In contrast, under the severe criteria of 3%/3 mm and 2%/2 mm, the gamma passing rate obtained for esophageal cases was higher than that found for NPC cases. Under all three criteria, the best passing rate was found for REC. Furthermore, the small SD shown in Fig. 2 (as indicated by the error bars) demonstrates that the Edose system is superior for the application of QA for IMRT. For further analysis, a comparison of the average gamma passing rates obtained with Edose compared with the TPS for the 30 IMRT cases is illustrated in Table 1.

**Table 1:** Comparison of average gamma passing rates obtained using Edose and TPS for 30 IMRT cases

Gamma criterion	Gamma passing rate (mean ± SD) %			
	NPC	EPC	REC	All treatment plans (30 cases)
5%/3 mm	(98.16 ± 0.90)%	(98.23 ± 0.75)%	(99.36 ± 0.61)%	(98.58 ± 0.93)%
3%/3 mm	(94.14 ± 1.45)%	(95.06 ± 1.28)%	(97.80 ± 0.84)%	(95.67 ± 1.97)%
2%/2 mm	(79.94 ± 1.81)%	(81.48 ± 3.63)%	(87.96 ± 3.06)%	(83.13 ± 4.53)%

### 3.B DVH comparison between Edose and TPS

In comparison to the TPS calculations, the ∆D_95_, ∆D_mean_, and ∆D_98_ of the PTVs obtained using Edose showed that the deviations were less than 3%, with the exception of the ∆D_98_ found for the PTV‐CTV in esophageal carcinoma patients, which was (3.21 ± 2.33)%, as shown in Table 2. The ∆D_95_ indicates higher value of (−2.05 ± 3.03)%, (−1.85 ± 2.87)%, (−2.00 ± 2.29)%, and (−2.50 ± 0.95)% for the PTV‐GTV‐N‐R and PTV‐GTV‐N‐L in NPC cases and the PTV‐CTV in EPC and REC cases, respectively. The same results were also obtained for ∆D_98._ However, larger deviations were found for the OARs than the targets. For example, in NPC plans, the values of ∆D_max_ were (−6.12 ± 5.28)% and (−2.41 ± 6.82)% for eye‐lens‐L and optic‐nerve‐L, respectively. The large SD indicated significant differences between Edose and TPS during delivery of the treatment plan. The values of V_30_ for the parotid gland in NPC, V_5_ for the lung in EPC, ∆D_max_ for the spinal cord in NPC and EPC were (−5.91 ± 8.70)%, (−1.98 ± 4.89)%, (−5.64 ± 3.84)% and (1.11 ± 3.35)%, respectively. In other words, the values found for the deviation of these OARs were greater than ± 3%.

**Table 2:** Differences (mean ± SD)% in DVH parameters of PTVs and OARs between the Edose reconstructions and TPS calculations of pretreatment measurements for 30 IMRT cases

Evaluated Organs	Edose vs. TPS				
	∆D_95_ (%)	∆D_mean_(%)	∆D98 (%)	∆D_max_ (%)	∆V_x_ (%)
NPC					
PTV‐GTV	−0.96 ± 1.38	0.19 ± 1.05	−1.37 ± 1.98		
PTV‐GTV‐N‐R	−2.05 ± 3.03	−0.27 ± 1.35	−2.66 ± 4.56		
PTV‐GTV‐N‐L	−1.85 ± 2.87	−0.15 ± 1.27	−2.19 ± 4.35		
PTV‐CTV1	−0.83 ± 1.39	−0.46 ± 1.41	−0.40 ± 1.35		
PTV‐CTV	−2.20 ± 1.60	0.02 ± 1.00	−2.97 ± 1.40		
PTV‐CTV‐N‐L	−0.85 ± 2.18	−0.72 ± 1.70	−0.34 ± 3.37		
PTV‐CTV‐N‐R	−0.83 ± 2.21	0.42 ± 1.40	−0.75 ± 1.82		
Brain‐stem				−3.63 ± 2.69	
Spinal				−5.64 ± 3.84	
Eye‐Len‐L				−6.12 ± 5.28	
Eye‐Len‐R				0.25 ± 8.73	
Parotid ‐L		−4.31 ± 3.59			−5.91 ± 8.70^c^
Parotid ‐R		0.31 ± 3.40			1.34 ± 6.52^c^
Optic‐nerve‐L				−2.41 ± 6.82	
Optic‐nerve‐R				−0.76 ± 6.16	
Optic‐chiasma				−3.86 ± 4.65	
EPC					
PTV‐GTV	−1.51 ± 2.35	0.63 ± 2.14	−2.10 ± 3.44		
PTV‐CTV	−2.00 ± 2.29	0.27 ± 1.16	−3.21 ± 2.33		
LUNG‐L					(−1.98 ± 4.89)^a^/(−2.59 ± 5.01)^b^
LUNG‐R					(−0.09 ± 3.36)^a^/(−1.70 ± 4.97)^b^
Spinal				1.11 ± 3.35	
REC					
PTV‐GTV	−0.46 ± 0.94	0.79 ± 0.92	−0.43 ± 1.14		
PTV‐CTV	−2.50 ± 0.95	−0.99 ± 0.56	−2.55 ± 1.94		
Small bowel				−1.34 ± 2.18	
Femoral head‐L		−3.07 ± 3.42			−2.28 ± 3.16^b^
Femoral head‐R		−0.95 ± 2.26			−1.59 ± 2.46^b^
Bladder		−3.55 ± 2.23			−6.12 ± 4.50^d^

a indicates ∆V_5_.

b indicates ∆V_20_.

c indicates ∆V_30_.

d indicates ∆V_40_.

**Figure 3:**
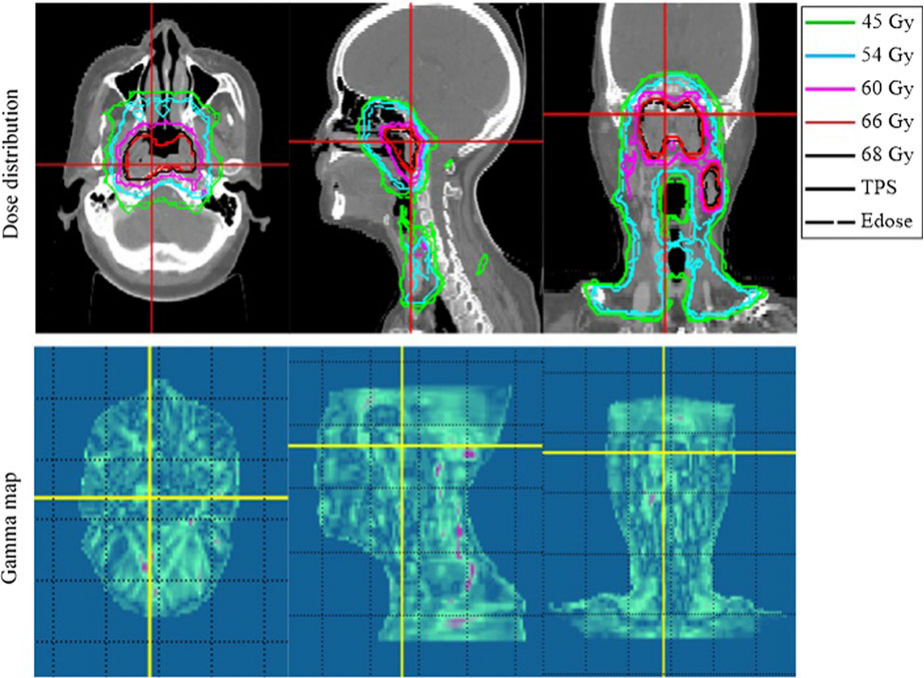
Comparison of dose distributions in axial, coronal and sagittal views obtained using Edose (dotted lines) and calculated using the TPS (solid lines) for one of the NPC patients. The gamma passing rate using the 3%/3 mm global criterion was 96.8%. The green isodose line represents the 45‐Gy distribution, the blue isodose line represents the 54‐Gy distribution, the pink isodose line represents the 60‐Gy distribution, the red isodose line represents the 66‐Gy distribution, and the black isodose line represents the 68‐Gy distribution.

**Figure 4:**
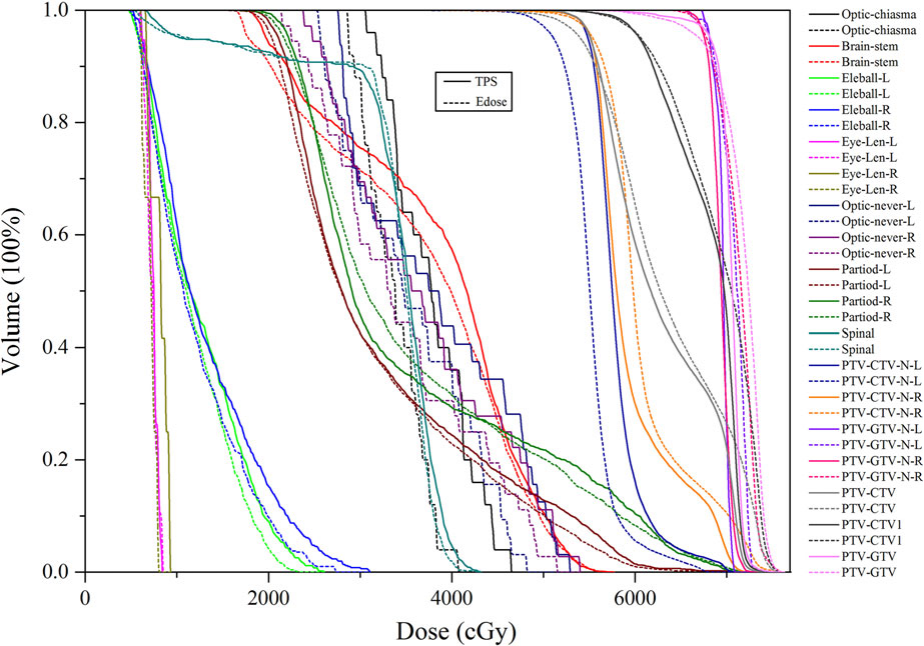
DVH comparisons of PTVs and OARs between Edose and TPS for one of the NPC patients. The solid lines represent the results of the TPS calculations, whereas the dotted lines represent the results of the Edose reconstruction. GTV: gross tumor volume; CTV: clinical target volume; CTV1: lymph nodes with a high risk of occult metastases; GTV‐N‐L: left GTV of neck lymph nodes; GTV‐N‐R: right GTV of neck lymph nodes; CTV‐N‐L: left of lymph nodes with low risk of occult metastases; CTV‐N‐R: right of lymph nodes with low risk of occult metastases. The PTV‐GTV, PTV‐GTV‐N‐L, PTV‐GTV‐N‐R, PTV‐CTV, PTV‐CTV1, PTV‐CTV‐N‐L, and PTV‐CTV‐L‐R were created by adding 5 mm to the margins of GTV, GTV‐N‐L, GTV‐N‐R, CTV, CTV1, CTV‐N‐L and CTV‐L‐R, respectively.

Using the NPC plan as an example, Fig. 3 shows the 3D dose distributions in three axial slices, and Fig. 4 shows the DVH comparison of the PTVs and OARs obtained using the Edose and TPS. As shown in Fig. 3, only small differences were detected in the dose distributions calculated with the TPS and reconstructed with the Edose system except in the cavity and bone regions. The solid lines represent the results of the TPS calculations, whereas the dotted lines represent the results of the Edose reconstruction. The 3D gamma analysis revealed that passing rate with the 3%/3 mm criterion was 96.8%. Overall, for all plans in this paper, the dose distributions had an excellent agreement between the TPS original plans and the reconstructed by Edose. The slice‐by‐slice comparisons revealed no significant dose differences.

## 4 DISCUSSION

Previous studies^16^, ^17^, ^18^ have indicated that Delta4 is superior in IMRT and VMAT patient pretreatment quality assurance. Therefore, Delta4 was selected in this study to compare the gamma analysis for the clinical cases with that reconstructed using Edose. The comparisons performed in this study showed that the Edose system could be suitably applied for clinical dose verification. The results showed that 3D dose verification (Edose) can provide not only the gamma passing rate but also the dose–volume relationship for the PTVs and OARs. In addition, the results show that Edose provides more detailed information and more accurate data for QA (such as the DVH of PTVs and OARs) in clinical radiotherapy. As a result, this system could further improve the QA of IMRT plans. In this study, the gamma analysis included comparisons only with Delta4 but did not compare the DVHs for two reasons. First, the CT image size of the Delta4 phantom is smaller than the patient CT image size, which becomes a problem when the target areas and OARs from the patient plan are imported into the Delta4 QA plan because the volume could change. The second reason is that the IMRT plans were created based on real patient CT images and had to be recalculated based on an artificial CT scan of the Delta4 phantom for dose verification. As the devices of the dose verification for IMRT, the Edose system and delta 4 device are based on different CT images.

Figures 1 and 2 summarize the results of the comparison of Edose and Delta4 as the treatment planning system, respectively. The gamma passing rate reached 92% under the criteria of 5%/3 mm and 3%/3 mm. Currently, a gamma passing rate of at least 88% under 3%/3 mm is widely used to indicate the feasibility of IMRT plans, 25 and many radiotherapy central believe that the gamma evaluation method is reliable and effective for IMRT treatment verification. The results reflect in Fig. 2 show that Edose is more suitable for QA of IMRT due to its small deviation and high stability.

Table 1 also shows that the gamma passing rate obtained for NPC cases is lower than that obtained for EPC and REC. Several factors might explain these results: (a) More high‐dose gradient regions, which were created in response to multiple critical organ dose limits in the vicinity of the tumor, were found in the NPC plan compared with the EPC and REC plans. (b) The NPC cases revealed more complicated, nonuniform CT anatomic structures than the EPC and REC cases. (c) Differences in the dose calculation for nonuniform anatomic structures were found between the CCCS algorithm and the AAA algorithm.^26^, ^27^ The calculation errors might be generated in Edose (version 3.01) during implementation of the dose algorithm and LINAC dose delivery. Thus, with the newer Edose version, we could improve our independent verification methods and separate the different sources of inaccuracy to improve the accuracy of the results.

The analysis of DVHs (as shown in Table 2) indicated that the differences in target areas were small, and larger dose deviations were found in small‐volume OARs. For example, the dose deviations obtained for the lens, parotid gland, optic nerve, and lungs in EPC and for the spinal cord exceeded 3% and even reached 6%. Furthermore, the larger SD of the OARs indicated that OARs are more sensitive than PTVs to changes in dosage. This pattern, which is illustrated in Fig. 4, was obtained because these OARs have a smaller volume; smaller dose differences would lead to a larger dose deviation in these OARs.^28^ The same results were obtained in a previous study conducted by Chen.^29^ The gamma passing rate is not sufficient for evaluating the feasibility of an IMRT plan, because it cannot accurately reflect the dose differences in the target areas and OARs of the anatomical structures of patients.^30^, ^31^ Thus, the highest safe doses for sensitive and important OARs should be adjusted according to the relationship between dose and anatomical structures.

As shown in Table 2, the deviation between the Edose reconstruction and the TPS calculation was greater than 5%, and this value was obtained for the clinical target areas located close to the skin surface, such as the PTV‐GTV‐N‐R in NPC. This phenomenon is related that the dose distribution is reconstructed using the fluence maps based on EPID. The dose of the built‐up region mainly come from the local dose deposition and relatively small contribution from the distal area, which would lead to an inability of the 3D dose verification system (Edose) to amend the dose to the built‐up region. In general, only a slight effect on the dose verification accuracy for the IMRT plan was found because the target areas were not too close to the built‐up area. However, a certain effect on *in vivo* measurements could be obtained, because the EPID as a detector for photon rays in the Edose system showed a low‐energy response.^32^ Therefore, subsequent versions of Edose should correct the scatter in the built‐up area to achieve better dose verification results.

## 5 CONCLUSIONS

This study tested a preliminary clinical application of 30 IMRT plans, and the results showed that the EPID‐based 3D dose verification system (Edose) was a simple and convenient QA tool for IMRT pretreatment dose verification. The gamma analysis included comparisons with the Delta4 system to validate the accuracy and reliability of the Edose system. The system could provide more clinical data and information through a single measurement. Furthermore, this system could allow a more intuitive and effective assessment for pretreatment plan dose verification. In future work, we would apply more verification tools to compare and verify the EPID‐based system, and proceed more detailed verification and clinical applicability of this system.

## ACKNOWLEDGMENTS

The authors are grateful to Raydose Medical Technology Co., Ltd for providing their EDose system and the technical support of computer.

## CONFLICT OF INTEREST

The authors have no conflicts of interest to disclose.
